# Acute kidney injury

**DOI:** 10.5249/jivr.v7i1.604

**Published:** 2015-01

**Authors:** Daniel Patschan, Gerhard Anton Müller

**Affiliations:** ^a^Department of Nephrology and Rheumatology, University Medical Center of Göttingen, Göttingen, Germany.

**Keywords:** Acute kidney injury, Pathophysiology, Therapy

## Abstract

Acute kidney injury is a frequent and serious complication in hospitalized patients. Mortality rates have not substantially been decreased during the last 20 years. In most patients AKI results from transient renal hypoperfusion or ischemia. The consequences include tubular cell dysfunction/damage, inflammation of the organ, and post-ischemic microvasculopathy. The two latter events perpetuate kidney damage in AKI. Clinical manifestations result from diminished excretion of water, electrolytes, and endogenous / exogenous waste products. Patients are endangered by cardiovascular complications such as hypertension, heart failure, and arrhythmia. In addition, the whole organism may be affected by systemic toxification (uremia). The diagnostic approach in AKI involves several steps with renal biopsy inevitable in some patients. The current therapy focuses on preventing further kidney damage and on treatment of complications. Different pharmacological strategies have failed to significantly improve prognosis in AKI. If dialysis treatment becomes mandatory, intermittent and continuous renal replacement therapies are equally effective. Thus, new therapies are urgently needed in order to reduce short- and long-term outcome in AKI. In this respect, stem cell-based regimens may offer promising perspectives.

## Introduction

Acute kidney injury (AKI) is still a major problem of today´s clinical medicine. It occurs in approximately 1-5% of all patients treated at the hospital. ^1^ The incidence significantly increases with progressive severity of the underlying cause: up to 50% of the patients treated at the intensive care unit develop AKI, in many cases as a results of generalized infection or sepsis. ^2^ The prognosis has not significantly been improved during the last 20-30 years, although substantial progress has been achieved in intensive care medicine and dialysis treatment, respectively. In the mid-nineteen seventies 70% of all patients with AKI died. Mortality moderately decreased until the early nineties (30-50%) and remained stable over the last 20 years. ^2^ The poor prognosis partly results from the disease leading to AKI per se but also ensues from complications associated with AKI. Thus, to establish more potent therapeutic interventions remains a fundamental goal in the field of nephrological research.

AKI is defined as acute deterioration of kidney function, as reflected by a significant increase in serum creatinine. In most patients (70%) urine output is reduced as well. The definition of the syndrome is periodically refined and according to the latest KDIGO-Guidelines, AKI can be diagnosed if the following criteria are fulfilled: (I) a serum creatinine increase of greater than 0.3 mg/dl within 48 hours, or (II) a 1.5-fold serum creatinine increase within seven days (as compared to a known or suspected baseline value), and / or (III) a reduction in urine output to less than 0.5 ml/kg/day for at least 6 hours. ^3^ It may not be forgotten that serum creatinine is a poor parameter of renal function since its concentration begins to rise late in AKI, which is if 60% of kidney function are lost. ^4^ New diagnostic markers are permanently being evaluated^4-6^ but shall not be reviewed in detail at the moment. The severity of AKI varies and there are several scores allowing to differentiate certain degrees of acute renal dysfunction. The RIFLE criteria distinguish between risk (R), injury (I), failure (F), loss (L), and end-stage renal disease (E), depending on the relative increase in serum creatinine and/or depending on the relative decrease in urine output^7,8^(Table 1). It has to be noted that stage E can only be diagnosed if (post)acute renal dysfunction persists for more than 3 months. ^9^ Such criteria are not necessarily relevant in therapeutic but in prognostic terms since the prognosis significantly declines with progressive severity of renal damage. ^7^

**Table 1:** RIFLE criteria.

Stage	Creatinine / GFR	Urine output
**R (isk)**	**1.5-fold increase** in serum creatinine / GFR reduction of 25% or more	**less than 0.5** ml/kg/h for at least **6 hours**
**I (njury)**	**2-fold increase** in serum creatinine / GFR reduction of 50% or more	**less than 0.5** ml/kg/h for at least **12 hours**
**F (ailure)**	**3-fold increase** in serum creatinine / GFR reduction of 75% or more	**less than 0.3** ml/kg/h for at least **24 hours**
**L (oss)**	**persistent renal failure** (after week 4)	
**E (SRD)**	**chronic kidney disease** (after month 3)	

**Etiology**

From a mechanistic point of view it has always been reasonable to distinguish three major causes of AKI. Any disease associated with obstruction of the urinary tract potentially induces post-renal AKI: hematoma within the renal pelvis or the ureter, tumors of the ureter or abdominal malignancies compromising urinary flow, urolithiasis, and diseases of the bladder and prostate to name the most important entities (Table 2). Together, they account for only 5% of AKI. In fact, the most frequent cause of the syndrome is transient renal hypoperfusion. In 55% AKI results from a significant decrease in mean arterial blood pressure (pre-renal AKI). Subsequently, several endogenous mechanisms are activated, intended to stabilize the intravascular blood volume and flow. ^10^ Among those are increased production of aldosterone and adiuretin, both decreasing renal excretion of water and sodium thereby reducing urine output. In more severe cases serum creatinine arises and AKI can be diagnosed. However, kidney function and structure are completely intact. The number of diseases responsible for pre-renal AKI is countless (e.g. heart failure of various origin, fluid and blood-losses, sepsis, intensified antihypertensive therapy Table 2), but the common characteristic is a reduction in effective arterial perfusion pressure. Pre-renal AKI is reversible as long as the cause has been eliminated and the tissue has not been structurally damaged. Intra-renal AKI (45%) displays two qualities that define the diagnosis: it does not result from urinary tract obstruction and the structure or microscopic architecture of the kidney is significantly altered. ^11^ In order to understand the etiology one has to realize that the kidney consists of four individual although functionally interdependent compartments: the glomerulum, the tubule, the vasculature, and the interstitium. Each compartment can be affected in a way that AKI ensues. More specific causes of intra-renal AKI are summarized in Table 2. AKI due to tubular damage accounts for 60% of intra-renal acute kidney injury. Morphologically, the cells display certain changes in their phenotype including epithelial cell flattening, loss of brush-border, nuclear loss, and apoptosis / necrosis. ^12^ The latter justifies the terminology acute tubular necrosis (ATN). ^13^ ATN can either result from endogenous / exogenous substances that deteriorate tubular cell metabolism followed by apoptosis / necrosis (Table 2) or it is induced by severe renal hypoperfusion. Thus, kidney ischemia initially causes pre-renal AKI and, depending on the duration of hypoperfusion and the individual tolerance of the tissue ischemic ATN if the underlying etiology / disease is not accurately being eliminated / treated. It is actually possible to differentiate between pre-renal AKI and ischemic ATN in some patients but this shall be explained later.

**Table 2:** Etiology of AKI. Pre-renal AKI accounts for 55%, while intra-renal AKI is being diagnosed in 45% of all patients. Post-renal AKI occurs rarely with 5%.

pre-renal	arterial hypotension	heart failure, fluid-loss, intensified anti-hypertensive treatment
		**infections:** postinfectious (various bacteria, less frequent: viruses, fungi)
	acute glomerulonephritis	**autoimmune-mediated diseases:** e.g. systemic lupus erythematosus, purpura schenlein-henoch, essential mixed cryoglobulinemia, anti-GBM syndrome, granulomatosis with polyangitis, microscopic polyangitis
		**idiopathic:** IgA-Nephropathy, idiopathic rapid progressive GN
		**drugs:** beta-lactams, proton pump inhibitors, allopurinol, NSAID
**intra-renal**	acute tubuluinterstitial nephritis	**infections:** e.g. leptospirosis, streptococcus, EBV
		**electrolyte / metabolic disorders:** hypokalemia, hyperuricemia, hypercalcemia
		**autoimmune-mediated diseases:** systemic lupus erythematodes, sjögren´s syndrome
	acute vasculopathy	renal artery embolism, renal vein thrombosis, thrombotic microangiopathy, renal crisis in systemic sclerosis
		**induced by tubulotoxic drugs:** aminoglycosides, cidofovir, foscarnet, vancomycin
	acute tubular necrosis	**induced by renal ischemia:** blood- / fluid-loss, schock of various origin
**post-renal**	urinary tract obstruction	hematoma of renal pelvis / ureter, malignancies (bladder, ureter, intestine, uterus), neurological disorders

**Pathogenesis of ischemic intra-renal AKI **

Since ischemic ATN or ischemic intra-renal AKI represents the most frequent type of AKI at the intensive care unit, the pathogenesis of the syndrome shall be discussed more in detail in this section.

Renal ischemia fundamentally affects the function and structure of the tubular epithelium. Nevertheless, two further events take place and are highly important for the dynamics of post-ischemic kidney regeneration: (I) interstitial inflammation and (II) microvasculopathy. ^11^ These three elements contributing to / prolonging kidney dysfunction after ischemia will be addressed separately.

**Tubular cell dysfunction and damage**

The tubular epithelium can significantly be impaired by ischemia which is reflected by both, functional and structural alterations. Ischemia decreases cellular production of ATP, resulting in a number of events severely disturbing the tubular homeostasis.^14^ The loss of ATP increases cytoplasmic calcium load which activates proteases, phospholipases, and caspases. ^15^ Thus, certain proteins and cell membrane-embedded phospholipids are degraded / destabilized. In addition, the cells get apoptotic. ^16^ Further consequences of ATP depletion are cellular accumulation of hypoxanthin and reactive oxygen species, further aggravating cell damage. ^15^ An early structural manifestation of ischemia is the loss of cell polarity with decreased reabsorption of sodium and water from the tubular lumen. Loss of polarity results from destabilization of cell-cell contact areas which structurally consist of Zonulae adherentes and occludentes. ^17^ Other morphological abnormalities include epithelial cell flattening, loss of brush-border, and nuclear loss. ^18^ Finally, epithelial cells detach from the basement membrane and accumulate within the tubular lumen. Such cell cluster inhibits the tubular flow and disintegration of the tubule promotes filtrate backflow into the interstitial space of the organ. Due to diminished sodium re-absorption, distal segments of the tubule are activated to release signals that induce constriction of the so-called Vasa afferentia (tubulo-glomerular feedback). ^10^ Together, the glomerular filtration rate decreases further. It has to be noted, that the terminology acute tubular necrosis is misleading since the dominant pattern of tubular cell damage is apoptosis and not necrosis. ^11^ Nevertheless, in severe cases of ischemic AKI the whole renal cortex may appear necrotic. Such manifestations can be seen in pregnancy-associated AKI or in septic shock. ^19^

**Inflammation**

Post-ischemic inflammation contributes to tissue damage in AKI although inflammatory processes are involved in kidney repair as well. As pointed out earlier, the outer medulla displays the highest vulnerability towards ischemia-induced damage. Inflammatory processes in this compartment partly result from endothelial upregulation of certain cell adhesion molecules such as intercellular adhesion molecule 1 and 2 (ICAM-1 and -2), CD99, and proteins of the junctional adhesion molecule family (JAM). ^20^ Subsequently, distinct leukocytes adhere to the endothelium which further aggravates tissue ischemia (vascular congestion). A diverse range of proinflammatory cytokines is released by tubular epithelial and vascular endothelial cells, inducing and perpetuating inflammation. ^11,21,22^ In this situation Il-6 and IRF-1 (interferon regulatory factor-1) most likely play key roles. Increased serum Il-6 levels for instance have been shown to predict mortality in AKI. ^23,24^ IRF-1 is being released by S3 tubular epithelial cells as an early response to ischemia. ^25^ and IRF-1 knockout animals show less ischemia-vulnerability as compared to their respective controls. ^26^ More or less all types of immune cells are activated in ischemic AKI including neutrophils, B cells, CD4+ T cells, macrophages, and NK cells. ^27^ Studies performed during the last 10-15 years examined the role of each of these cell populations in ischemic AKI, the results were often conflicting. ^27^ Blocking neutrophils was successful in one study but protective effects were absent in other investigations. ^28^ Macrophages infiltrate the post-ischemic kidney to a significant extent and they have been shown to contribute to renal fibrosis in AKI. ^29^ On the other hand, macrophages have also been documented to transdifferentiate into an anti-inflammatory M2 phenotype, promoting renal repair after ischemia^27^ Several interesting studies were performed regarding the role of T cells in AKI. Among this population, CD4+ cells are apparently an essential element in the process of ischemia-induced damage. Selective CD4 but not CD8 cell depletion protect mice from AKI and administration of the cells restores ischemia-vulnerability.^30^ Additional investigations revealed that particularly Th1 CD4+ cells represent the pathogenic relevant cohort and that their activity critically depends on production / secretion of Il-16 by tubular epithelial cells. ^31^ Thus, it becomes evident that postischemic inflammation is essential in terms of aggravating tissue damage and mediating tissue repair in AKI.

**Microvasculopathy**

The relevance of postischemic microvasculopathy has been proven for the first time in the early nineteen-seventies. Mannitol treatment of animals abrogated postischemic endothelial cell swelling in the kidney, subsequently promoting faster reperfusion and tissue regeneration. ^32^ Comparable observations were made by Prof. M. Goligorsky from the New York Medical College. ^33,34^ Nude rats subjected to renal ischemia displayed endothelial cell swelling in intrarenal capillaries, associated with slower postischemic reperfusion. Such no-reflow phenomenon was partly reversible if the animals were injected with mature endothelial cells of human origin. Thus, cells of the endothelial lineage acted renoprotective by modulating the vascular structure / function. Since then, further studies expanded this therapeutic approach. During the last 7 years, so-called Endothelial Progenitor Cells (EPCs) were successfully administered in murine AKI. ^35,36^ In addition, several protocols have been established for increasing the cells´ renoprotective competence prior to injection. ^18,37-40^ Another aspect of postischemic microvasculopathy is related to the risk for developing chronic renal failure in the long-term. Morphological analyses of kidneys from animals with AKI reveal a decrease in peritubular vascular density with increased accumulation of connective tissue in the interstitial space. ^41,42^ Interstitial fibrosis indicates a higher risk for chronic tissue damage and current investigations focus on the role of vascular rarefication as risk factor for chronic kidney disease per se.

In summary, ischemic ATN is not an exclusively tubular disease but also involves the interstitial space and the vasculature. Damages within / of the two latter compartments can significantly perpetuate kidney malfunction in AKI.

**Clinical manifestations**

Patients with AKI do not suffer from clinical symptoms more or less specific for the disease. On one hand, they may present manifestations of the underlying disease (e.g. heart failure, sepsis, systemic vasculitis, thrombotic microangiopathy). If renal function is truly affected the typical course of AKI includes 4 stages: (I) initiation, (II) oligo-anuria, (III) polyuria, and (IV) restitution. In this dynamic process, clinical signs of renal dysfunction emerge during stage 2 (oligo-anuria). Urine output is diminished in 70% of AKI ^43^ and the consequences may involve fluid retention with aggravated hypertension and heart failure with pulmonary edema. Due to diminished excretion of electrolytes and endogenous / exogenous waste products, the whole organism is affected. The term uremia describes such toxification and it is associated with diverse and heterogenous symptoms including pruritus, neurological manifestations, nausea and vomiting, diarrhea, loss of appetite with anorexia, cardiac arhythmia, and insomnia. In addition, the patients have a higher risk for infection and bleeding complications (disturbed thrombocyte function). The presence of uremia is important since in most cases dialysis treatment becomes mandatory. Stage 3 (polyuria) usually indicates beginning recovery of kidney function but it can cause significant losses of water, sodium and potassium. The latter may induce cardiac arhythmia. If the process of renal recovery lasts longer than 3 months, AKI has been transformed into chronic kidney disease or CKD. ^44^

**Diagnosis and differential diagnosis**

The approach to the patient with suspected AKI includes several diagnostic steps. The first question must be related to urinary tract obstruction. Despite the fact that post-renal AKI accounts for only 5%, ultrasound analysis of the kidney, ureter, and bladder is the first diagnostic measure. The next two steps are intended to identify (I) renal hypoperfusion and (II) drug-induced AKI. It has to be evaluated whether the patient´s history indicates any episodes of hypotension during the last days. The number of drugs potentially causing AKI is high but the most important substances should be known by every physician (aciclovir, aminoglycosides, NSAID, sulfonamides, and vancomycin). Then, urine and blood analyses should be performed. Urine analysis can reveal acute glomerulonephritis, tubulointerstitial nephritis, and in some cases it allows to discriminate pre-renal from ischemic intra-renal AKI (Table 3). Certain blood parameters may become important in patients with suspected autoimmune-mediated disease (Table 3). Other diagnostic steps are usually not necessary unless the diagnosis is still questionalbe. By ultrasound blood-flow measurements or NMR of the renal vasculature, renal artery embolism or renal vein thrombosis may be diagnosed. Finally, some patients may require renal biopsy in order to find the relevant cause of AKI. Table 3 summarizes the diagnostic steps leading to the etiology.

**Table 3:** Step-wise approach to the patient with AKI. It has to be noted that ultrasound analysis is mandatory although post-renal AKI can be diagnosed in only 5% of all patients.

diagnostic step	procedure	comments
**1**	**ultrasound**	**obstruction?**
**2**	**hemodynamic evaluation**	history of **hypotension** during the last days ?
**3**	**drug history**	aciclovir, aminoglykosides, NSAID, sulfonamides, vancomycin
		• erhytrocyte casts, acanthocytes: **acute glomerulonephritis**
**4**	**urine** analysis	• eosinophiluria, tubular proteinuria: **interstitial nephritis**
		• urine osmolality: <350 mosmol/kg – **ATN**, >500 mosmol/kg – **pre-renal AKI**
		• ANA, anti-dsDNA, hypocomplementemia – **SLE**
		• cANCA / pANCA – **GPA, MPA**
**5**	**blood analysis**	• anti-GBM – **acute glomerulonephritis**
		• cryoglobulins - **acute glomerulonephritis**
		• thrombocytopenia, anemia, LDH increase – **thrombotic microangiopathy**
		• myoglobin / hemaglobin – **rhabdomyolysis / hemolysis**
**6**	**imaging**(ultrasound, NMR)	**renal artery embolism, renal vein thrombosis**
**7**	**renal biopsy**	in cases of **unknown etiology**

**Therapy and outcome**

The prognosis of AKI is still poor. As pointed out earlier, between 30 and 50% of all AKI patients die despite treatment has been initiated.^1^ Such high mortality has two reasons: in many situations the prognosis is significantly determined by the disease leading to AKI. For instance, sepsis is a generalized inflammatory syndrome, affecting kidney, lung, heart, brain, and the immune system. ^45^ Second, no renal replacement therapy regimen is equivalent to the normal kidney in terms of detoxification. The impact of the latter is illustrated by the prognosis of CKD patients undergoing dialysis treatment on a regular basis. The mean life-span of dialysis patients is 10 years, the mean life-span in transplant recipients ranges from 20-25 years . It becomes evident that the fundamental goal remains to establish therapies that promote the recovery process of the kidney tissue per se.

At the moment, therapy of AKI includes three goals: (I) treatment of the disease associated with AKI, (II) minimizing further aggravation of kidney damage, and (III) treatment of complications. Measures needed to achieve the first goal depend on the etiology (e.g. fluid- / blood-administration, vasoactive substances in heart failure, antibiotics in sepsis). However, in many cases kidney damage has been evolved even if the initial cause has been eliminated. In order to achieve the second goal, drugs with must be avoided that typically affect the function / structure of the organ and in addition, the mean arterial blood pressure should be elevated to at least 65 mmHg. Regarding the third goal, some but by no means every complication(s) of AKI can be treated with certain medications (hyperkalemia, metabolic acidosis, and hyperhydration). Two problems remain unsolved so far. First, there are no drugs available today that truly stimulate the excretory function of the kidney. Second, the dynamics of kidney regeneration can't substantially be modulated by pharmacological regimens yet. Therefore, in many situations dialysis treatment becomes mandatory.

Renal replacement therapy must be initiated if the patient presents with refractory hyperhydration, hyperkalemia, and acidosis. In addition, if any uremia-associated symptoms are being diagnosed, dialysis therapy is indispensible. ^46^ It remains a matter of debate which dialysis regimen is superior in AKI. The two principal alternatives are intermittent and continuous renal replacement therapy. Intermittent dialysis is being performed every other day for several hours per treatment session. During this period fluid and waste products must be eliminated and this can be associated with hemodynamic destabilization in theory. Continuous dialysis on the other hand is a daily procedure with lower rates of fluid depletion from the body. However, no study showed any significant advantage for one procedure over the other. ^47^

In the past, the effectiveness of diverse drugs in preventing patients from AKI or in promoting tissue repair in AKI has been evaluated. Among the substances subjected to analysis were dopamine, calcium channel blocker, theophylline,^48^ adenosine, ^49^ and N-acetylcysteine (radiocontrast-induced nephropathy. ^50^) Nevertheless, none of these strategies truly improved the prognosis in AKI in the clinical setting. Regarding radiocontrast-induced nephropathy, the only measure with significant impact is intravenous administration of isotonic saline solution. In order to achieve renoprotective effects saline must be infused at a dose of 1 ml/kg/h, therapy should be started 12 hours prior to contrast media administration. ^51^ The currently available data does not show any benefit of dialysis treatment after contrast media administration. ^52^

In summary, AKI remains a fundamental problem in the hospital. The current therapy essentially focuses on the prevention of further kidney damage. Thus, new measures that substantially improve kidney regeneration are urgently needed. In this context, cell-based therapies may offer promising perspectives.
